# High-Flow Nasal Cannula oxygen therapy in COVID-19: retrospective analysis of clinical outcomes – single center experience

**DOI:** 10.3389/fmed.2023.1244650

**Published:** 2023-10-02

**Authors:** Dušanka Obradović, Aleksandra Milovančev, Aleksandra Plećaš Đurić, Stanislava Sovilj-Gmizić, Vladimir Đurović, Jovica Šović, Miloš Đurđević, Stevan Tubić, Jelena Bulajić, Milena Mišić, Jovana Jojić, Miroslava Pušara, Ivana Lazić, Mladen Đurković, Renata Bek Pupovac, Aleksandra Vulić, Marija Jozing

**Affiliations:** ^1^Faculty of Medicine Novi Sad, University of Novi Sad, Novi Sad, Serbia; ^2^Institute for Pulmonary Diseases of Vojvodina, Sremska Kamenica, Serbia; ^3^Institute for Cardiovascular Diseases of Vojvodina, Sremska Kamenica, Serbia; ^4^Clinic of Anesthesiology, Intensive Care and Pain Therapy, University Clinical Center of Vojvodina, Novi Sad, Serbia; ^5^Clinic of Nephrology and Clinical Immunology, University Clinical Center of Vojvodina, Novi Sad, Serbia; ^6^Urgent Care Center, University Clinical Center of Vojvodina, Novi Sad, Serbia

**Keywords:** COVID-19, acute hypoxemic respiratory failure, High-Flow Nasal Cannula oxygen therapy, clinical outcomes, intensive care unit

## Abstract

**Background:**

High-Flow Nasal Cannula (HFNC) oxygen therapy emerged as the therapy of choice in COVID-19-related pneumonia and moderate to severe acute hypoxemic respiratory failure (AHRF). HFNC oxygen therapy in COVID-19 has been recommended based its use to treat AHRF of other etiologies, and studies on assessing outcomes in COVID-19 patients are highly needed. This study aimed to examine outcomes in COVID-19 patients with pneumonia and severe AHRF treated with HFNC.

**Materials and methods:**

The study included 235 COVID-19 patients with pneumonia treated with HFNC. Data extracted from medical records included demographic characteristics, comorbidities, laboratory parameters, clinical and oxygenation status, clinical complications, as well as the length of hospital stay. Patients were segregated into two groups based on their oxygen therapy needs: HDU group, those who exclusively required HFNC and ICU group, those whose oxygen therapy needed to be escalated at some point of hospital stay. The primary outcome was the need for respiratory support escalation (noninvasive or invasive mechanical ventilation) and the secondary outcome was the in-hospital all-cause mortality.

**Results:**

The primary outcome was met in 113 (48%) of patients. The overall mortality was 70%, significantly higher in the ICU group [102 (90.2%) vs. 62 (50.1%), *p* < 0.001]. The rate of intrahospital infections was significantly higher in the ICU group while there were no significant differences in the length of hospital stay between the groups. The ICU group exhibited significant increases in D-dimer, NLR, and NEWS values, accompanied by a significant decrease in the SaO_2_/FiO_2_ ratio. The multivariable COX proportional regression analysis identified malignancy, higher levels of 4C Mortality Score and NEWS2 as significant predictors of mortality.

**Conclusion:**

High-Flow Nasal Cannula oxygen therapy is a safe type of respiratory support in patients with COVID-19 pneumonia and acute hypoxemic respiratory failure with significantly less possibility for emergence of intrahospital infections. In 52% of patients, HFNC was successful in treating AHRF in COVID-19 patients. Overall, mortality in COVID-19 pneumonia with AHRF is still very high, especially in patients treated with noninvasive/invasive mechanical ventilation.

## 1. Introduction

Coronavirus disease 2019 (COVID-19) pandemic changed the pathophysiological insights of many clinical symptoms and signs in the respiratory medicine field, especially relative to the impairment of the gas exchange induced by pneumonia due to the SARS CoV-2 virus (1). Acute hypoxemic respiratory failure (AHRF) was the main reason for hospital admission of patients with COVID-19 pneumonia. The required level of respiratory support depended on the extent of the lung parenchyma involved, comorbidities, and age. The incidence and outcomes of severe COVID-19 associated AHRF are influenced by various factors including indications for hospitalization during different pandemic waves, hospital organization, and available equipment (2, 3).

Early recognition of respiratory support failure is a crucial factor that can significantly impact the outcome of patients with COVID-19 and AHRF. During the COVID-19 pandemic, High Flow Nasal Cannula oxygen therapy (HFNC) was introduced as a noninvasive respiratory support for patients with COVID-19 pneumonia and AHRF. The positive effects of HFNC before the pandemic were first recognized in pediatric population (4). The use of HFNC for respiratory support in adults has been on the rise since the 2000s (5) and it was recommended in the guidelines even before the pandemic era. American College of Physicians (6) and European Society of Intensive Care Medicine (7) recommended the use of HFNC over noninvasive positive pressure ventilation (NIPPV) for treating AHRF. European Respiratory Society released the clinical guidelines for using High-Flow Nasal Cannula in acute respiratory failure implying that it had the advantage over Conventional Oxygen Therapy (COT) and NIPPV (8). In systematic review and meta-analysis (9), which included twenty five randomized clinical trials of patients with AHRF, all studies showed lower risk of intubation when the HFNC was used. In COVID-19, AHRF primarily occurs as a result of ventilation-perfusion shunt. In the majority of cases, this condition manifests clinically and radiologically as acute respiratory distress syndrome (ARDS). The benefits of HFNC in COVID-19 patients with AHRF vary across different studies. For instance, the randomized clinical trial conducted by the Ospina et al. (10) demonstrated advantages of HFNC over COT in terms of reduced intubation incidence and shorter time to recovery. However, the RECOVERY-RS trial (11) found no benefits of HFNC in terms of intubation and mortality rate. Our study aimed to examine HFNC outcomes in COVID-19 patients with pneumonia and severe AHRF.

## 2. Methodology

### 2.1. Study population and selection criteria

Our retrospective study included patients admitted to the tertiary-level care University Clinical Centre of Vojvodina at the dedicated COVID facility “Mišeluk,” from October 2021 to April 2022 with following eligibility criteria: ≥18 years of age, SARS-CoV-2 detected in nasopharyngeal swab using real-time reverse transcription-polymerase chain reaction assay, clinical and radiological signs of pneumonia (bilateral and peripheral ground-glass opacities and consolidations) (12), SpO2 < 94% on room air at sea level, respiratory rate > 30 breaths/min (13) and SaO_2_/FiO_2_ ratio < 315 mmHg. SaO_2_/FiO_2_ was used as a surrogate of PaO_2_/FiO_2_ ratio due to resource limitations, as it was recently proposed as an alternative criterion for acute respiratory distress syndrome (ARDS) (14). This has been supported by several previous studies involving COVID-19 patients with severe AHRF (15–17). The exclusion criteria were: the use of any other respiratory support prior to HFNC, duration of the HFNC less than 24 h; patients with hypercapnic respiratory failure and respiratory acidosis who were treated consequently with NIPPV, those with clinical signs of shock and the ones who required immediate intubation were also excluded. According to the WHO Progression Scale, patients were classified as severe disease with score 6 (13) after a short course of COT (less than 2 h) without achieving the peripheral oxygen saturation ≥ 90% at oxygen flow rate ≥ 15 L/min using a facial mask or non-rebreathing mask and without resolving the signs of respiratory distress.

### 2.2. Treatment protocol and patient groups

During the abovementioned period, 1,033 patients with COVID-19 pneumonia and acute hypoxemic respiratory failure were admitted to the High Dependency Unit (HDU). HFNC was provided via mechanical ventilators (Prunus Medical Boaray 5000D, Shenzhen, Guangdong, China) using the high-flow oxygen therapy mode. The initial flow rate of 30 to 60 L/min, alongside FiO_2_ up to 100%, were subsequently adjusted to achieve a target oxygen saturation of ≥92% while reducing dyspnea and respiratory rate. There were two groups based on their oxygen therapy needs: those who exclusively required HFNC (HDU group) and those whose oxygen therapy needed to be escalated [NIPPV or invasive mechanical ventilation (IMV)] at some point of hospital stay (ICU group) due to the low SpO_2_ levels and signs of respiratory distress (tachypnea, dyspnea, usage of the auxiliary respiratory musculature). All patients were treated with either the standard of care (SOC) therapy or SOC along with baricitinib according to the National Institutes of Health guidelines for treatment of COVID-19 hospitalized patients (13).

### 2.3. Baseline, clinical, laboratory parameters and outcomes

Data were collected from electronic medical records and included demographic characteristics, comorbidities, vaccinal and smoking status, duration of illness prior to admission and laboratory parameters on admission, as well as the Coronavirus Clinical Characterization Consortium Mortality Score (4C mortality score) (18), neutrophil to lymphocyte ratio (NLR) (19) and National Early Warning Score 2 (NEWS2) (20). For the ICU group, levels of proinflammatory markers, D-dimer, NRL, as well as the values of the NEWS2, were reevaluated shortly prior to the patient’s transfer to the ICU. ROX index (21) values were not assessed upon admission due to resource constraints and the absence of an immediate clinical imperative. Instead, ROX index evaluation was conducted only before the patient’s transfer to the ICU when it became clinically relevant. We have also collected the data on frequency of intrahospital infections, pneumothorax/pneumomediastinum, pulmonary embolism, and the length of hospital stay. The primary outcome was defined as HFNC failure with the need for respiratory support escalation which included either noninvasive or invasive mechanical ventilation. The secondary outcome was the in-hospital all-cause mortality. The study protocol was approved by the Ethics Committee of the University Clinical Centre of Vojvodina (protocol code: 00-39, date of approval: 9 February 2023).

### 2.4. Statistical analysis

The normality of the continuous variables was assessed using the Kolmogorov–Smirnov test. Non-normally distributed continuous variables were reported as the median with the interquartile range (Q1-Q3). Continuous variables were compared between independent groups using the Wilcoxon rank-sum test, while paired samples were analyzed using the paired Wilcoxon rank-sum test. Categorical variables were compared using the chi-square test or Fisher’s exact test, as appropriate. Cox proportional hazards modeling was used to examine the association between the outcomes and predictor variables. Hazard ratios (HR) and their 95% confidence intervals (CI) were reported to quantify the magnitude and direction of the associations. Kaplan–Meier curves were used to visualize survival probabilities, stratified by relevant variables. All statistical tests were two-tailed and the alpha level of 0.05 was set as a significance threshold. No imputations were used for the missing data. Statistical analyses were conducted using RStudio 2023.03.1 + 446 “Cherry Blossom” Release.

## 3. Results

### 3.1. Baseline patients’ characteristics

**Figure 1:**
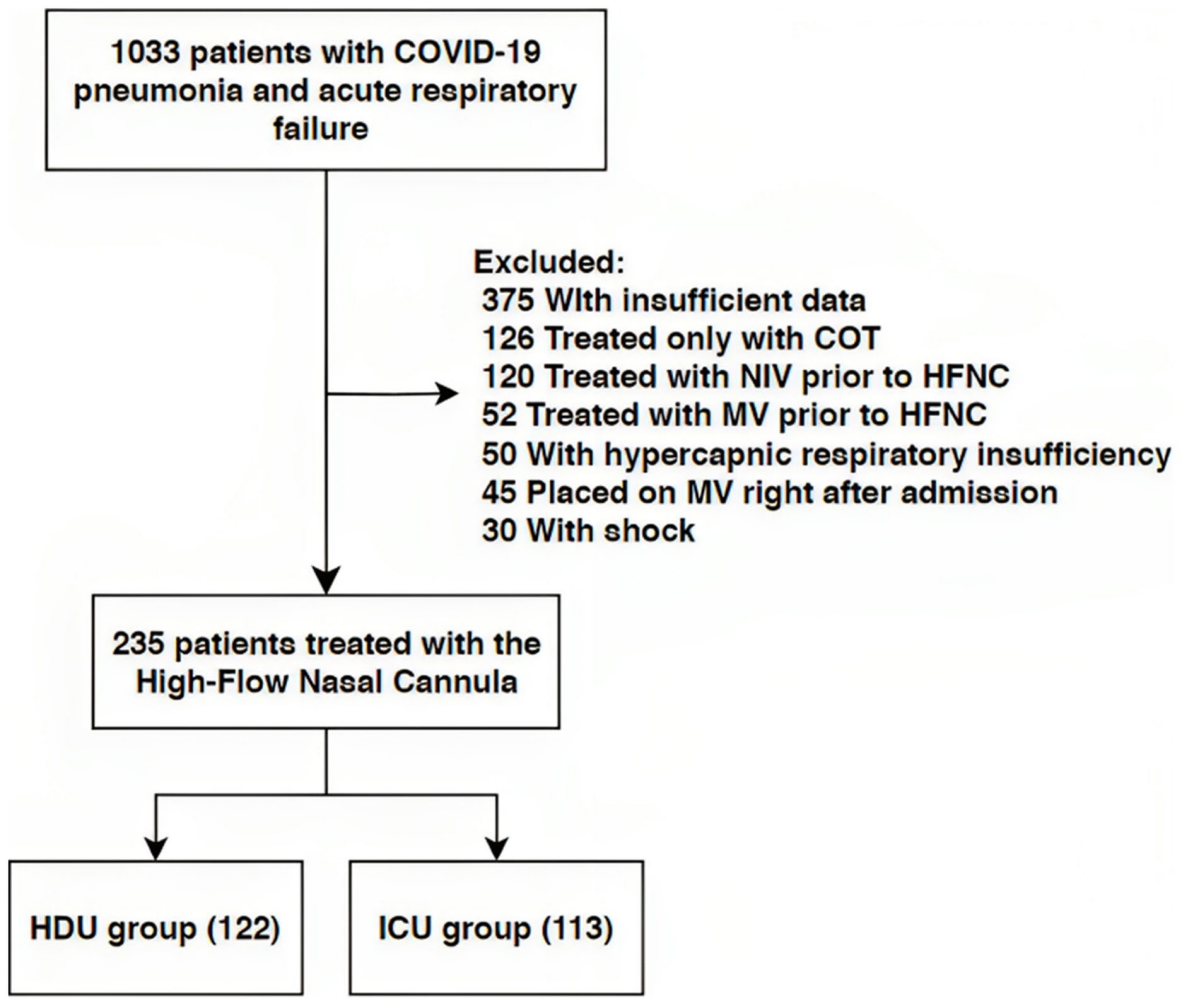
Flow chart of patient selection. NIV, Non-Invasive Ventilation; HFNC, High Flow Nasal Cannula; MV, Mechanical Ventilation; HDU, High Dependency Unit; ICU, Intensive Care Unit.

Our study included 235 patients treated with HFNC (Figure 1). The median age was 70 years (62–79) with 58.3% of males. Hypertension was the most common comorbidity, observed in 67.7%, with 10.6% smokers. The group of patients without the progression of respiratory failure were significantly older [72 years (61–84) vs. 70 (63–75), respectively, *p* = 0.022]. There were no significant differences in the prevalence of comorbidities between the two groups, nor were there any significant differences regarding vaccination status. Table 1 provides an overview of the baseline characteristics of the patients at hospital admission. There were also no significant differences in admission parameters between groups as shown in Table 2.

**Table 1:** Patients’ characteristics at the admission.

Parameter^1^	Overall, *N* = 235^2^	HDU, *N* = 122^2^	ICU, *N* = 113^2^	*p*^3^
Sex (*M*)	137 (58.3)	72 (59)	65 (57.5)	0.816
Age	70 (62–79)	72 (61–84)	70 (63–75)	**0.022**
Disease duration prior to admission (days)	8 (2–7)	7 (5–10)	8 (5–10)	0.691
Disease duration before the diagnosis (days)	5 (2–7)	4 (2–6)	5 (3–8)	**0.043**
Vaccinated	64 (27.2)	35 (28.7)	29 (25.7)	0.603
Smoker	25 (10.6)	13 (10.7)	12 (10.6)	0.993
Comorbidities				
Hypertension	159 (67.7)	83 (68.0)	76 (67.3)	0.899
Diabetes	75 (31.9)	37 (30.3)	38 (33.6)	0.588
Obesity	52 (22.1)	21 (17.2)	31 (22.4)	0.059
Cardiomyopathy	27 (11.5)	15 (12.3)	12 (10.6)	0.687
Atrial fibrillation	13 (5.5)	7 (5.7)	6 (5.3)	0.886
COPD	17 (7.2)	9 (7.4)	8 (7.1)	0.930
Asthma	14 (6.0)	5 (4.1)	9 (8.0)	0.211
CKD	13 (5.5)	9 (7.4)	4 (3.5)	0.199
History of stroke	13 (5.5)	8 (6.6)	5 (4.4)	0.475
Malignancy	24 (10.2)	16 (13.1)	8 (7.1)	0.127

1 COPD, Chronic obstructive pulmonary disease; CKD, Chronic kidney disease.

2 Median (Q1–Q3)/*n* (%).

3 Wilcoxon rank sum test; Pearson’s Chi-squared test.

**Table 2:** Admission parameters.

Parameter^1^	Overall, *N* = 235^2^	HDU, *N* = 122^2^	ICU, *N* = 113^2^	*p*^3^
CRP (mg/L)	130 (74–190)	132 (61–206)	121 (81–176)	0.969
PCT (ng/mL)	0.19 (0.09–0.37)	0.18 (0.08–0.34)	0.21 (0.11–0.53)	0.140
D-dimer (mg/L)	1.6 (1.0–3.2)	1.7 (1.0–4.3)	1.5 (0.9–2.7)	0.132
ALT (IU/L)	37 (24–56)	34 (22–55)	41 (27–59)	0.131
AST (IU/L)	48 (35–73)	46 (35–69)	51 (36–76)	0.414
GGT (IU/L)	53 (32–87)	52 (30–86)	56 (35–87)	0.551
Leukocyte count (× 10^9^)	8.7 (6.4–12.3)	8.7 (6.8–12.6)	8.7 (6.1–11.8)	0.399
Lymphocyte count (× 10^9^)	0.73 (0.48–1.03)	0.75 (0.47–1.05)	0.70 (0.48–1.01)	0.817
Neutrophil count (× 10^9^)	7.3 (5.1–10.4)	7.3 (5.6–10.5)	7.2 (4.7–10.1)	0.411
Thrombocyte count (× 10^9^)	194 (145–268)	203 (153–271)	182 (141–255)	0.160
Erythrocyte count (× 10^12^)	4.73 (4.27–5.12)	4.77 (4.26–5.15)	4.64 (4.27–5.12)	0.405
Hemoglobin (g/L)	134 (123–147)	135 (123–147)	133 (123–148)	0.914
SaO_2_/FiO_2_	1.19 (0.99–1.58)	1.17 (1.00–1.58)	1.34 (0.98–1.58)	0.586
NLR	9 (6–17)	9 (6–18)	10 (5–16)	0.686
NEWS2	6 (4–8)	6 (4–8)	6 (4–7)	0.530
4C Mortality Score	13 (10–15)	13 (10–15)	13 (10–15)	0.647

1 CRP, C-reactive protein; PCT, Procalcitonin; ALT, Alanine aminotransferase; AST, Aspartate aminotransferase; GGT, Gamma glutamyl transferase; NLR, Neutrophil-to-Lymphocyte Ratio; NEWS2, National Early Warning Score 2; 4C Mortality score, Coronavirus Clinical Characterization Consortium Mortality Score.

2 Median (Q1–Q3).

3 Wilcoxon rank sum test.

### 3.2. Intrahospital events and treatment measures

Among the patients included in the study, 18.7% acquired intrahospital infections during their hospital stay. The incidence of intrahospital infections was significantly higher in the group of patients who required non-invasive ventilation or mechanical ventilation [30 (26.5%) vs. 14 (11.5%) *p* = 0.003]. Specifically, patients in this group had a higher prevalence of urinary tract infections [16 (14.2%) vs. 3 (2.5%), *p* = 0.001] and intrahospital pneumonia was exclusively observed in this group [18 (15.9), *p* < 0.001] (Table 3). There were no significant differences between the two groups in terms of corticosteroid therapy, the use of baricitinib, or the occurrence of pulmonary thromboembolism, pneumothorax, and pneumomediastinum (Table 4).

**Table 3:** Intrahospital infections.

Parameter	Overall, *N* = 235^1^	HDU, *N* = 122^1^	ICU, *N* = 113^1^	*p*^2^
Intrahospital infections	44 (18.7)	14 (11.5)	30 (26.5)	**0.003**
Urinary tract infection	19 (8.1)	3 (2.5)	16 (14.2)	**0.001**
*Clostridium difficile* Infection	20 (8.5)	13 (10.7)	7 (6.2)	0.221
Intrahospital pneumonia	18 (7.7)	0 (0)	18 (15.9)	**< 0.001**

1 *n* (%).

2 Pearson’s Chi-squared test; Fisher’s exact test.

**Table 4:** Treatment and complications.

Therapy/complication	Overall, *N* = 235^1^	HDU, *N* = 122^1^	ICU, *N* = 113^1^	*p*^2^
Corticosteroids	227 (96.6)	115 (94.3)	112 (99.1)	0.067
Baricitinib	33 (14.0)	19 (15.6)	14 (12.4)	0.483
Pulmonary thromboembolism	22 (9.4)	11 (9.0)	11 (9.7)	0.850
Pneumothorax	12 (5.1)	6 (4.9)	6 (5.3)	0.892
Pneumomediastinum	13 (5.5)	4 (4.1)	8 (7.1)	0.318

1 *n* (%).

2 Pearson’s Chi-squared test.

### 3.3. Clinical course and outcomes

Patients who were transferred to the ICU did not exhibit a significant change in CRP and PCT levels. However, significant increases were observed in D-dimer, NLR, and NEWS2 values. Additionally, there was a significant decrease in SaO_2_/FiO_2_ ratio, reflecting worsening oxygenation. The median ROX score at the ICU transfer was 3.40 (2.80–4.55) as shown in Table 5.

**Table 5:** Laboratory parameters’ progression in the ICU group.

Parameter^1^	At admission, *N* = 113^2^	Transfer to the ICU, *N* = 113^2^	*p*^3^
CRP	121 (81–176)	117 (72–173)	0.683
PCT	0.21 (0.11–0.53)	0.21 (0.11–0.78)	0.288
D-dimer	1.53 (0.92–2.70)	2.07 (1.26–4.30)	**0.019**
NLR	10 (5–10)	14 (9–23)	**<0.001**
NEWS2	6 (4–7)	8 (6–10)	**<0.001**
SaO_2_/FiO_2_	1.34 (0.98–1.58)	0.94 (0.88–1.03)	**<0.001**
ROX index	–	3.40 (2.80–4.55)	**–**

1 CRP, C-Reactive Protein; PCT, Procalcitonin; NLR, Neutrophil-to-Lymphocyte Ratio; NEWS2, National Early Warning Score 2.

2 Median (Q1–Q3).

3 Wilcoxon signed rank test with continuity correction.

#### 3.3.1. Primary outcome

HFNC failure was observed in 113 patients (48%) with 96 of them (85%) initially receiving noninvasive ventilation. Among the participants who initially received noninvasive ventilation, 75 of them (78%) required subsequent mechanical ventilation. Patients that were placed in the ICU received HFNC oxygen therapy for the significantly shorter amount of time (2 days vs. 12 days, *p* < 0.001).

#### 3.3.2. Secondary outcome

**Figure 2:**
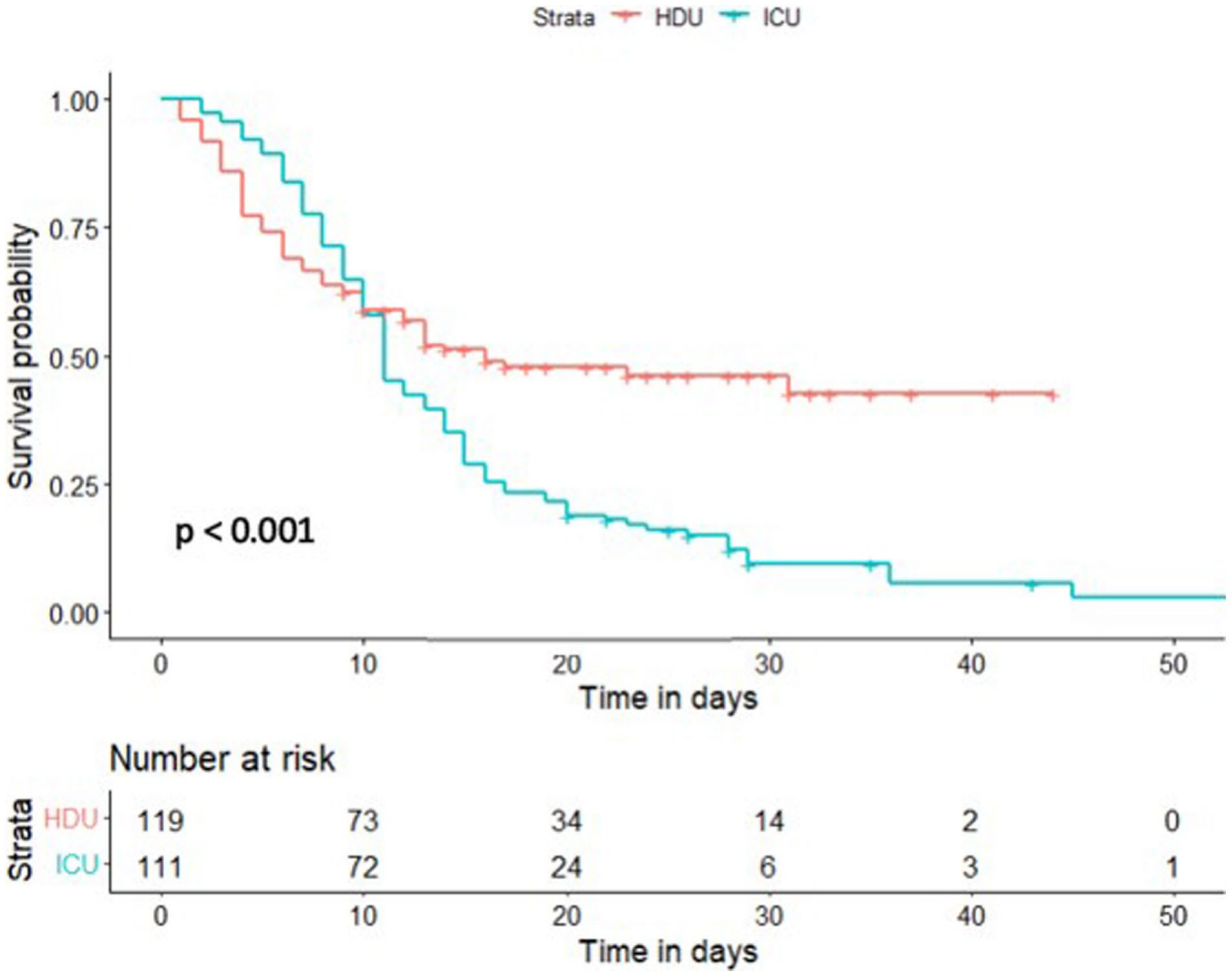
Kaplan–Meier estimate of survival of High Flow Nasal Cannula (HFNC) treated patients stratified by the primary outcome. HDU, High Dependency Unit; ICU, Intensive Care Unit.

Regarding the secondary outcome, which was observed in 164 patients (70%), a significantly higher mortality rate was reported in the ICU compared to the HDU group [102 (90%) vs. 62 (51%), *p* < 0.001]. The length of hospital stay did not differ significantly between the two groups as shown in Table 6. The HDU group exhibited a significantly better survival compared to the ICU group, with a hazard ratio (HR) of 0.56 (95% CI 0.40–0.77, *p* < 0.001), as demonstrated in Figure 2.

**Table 6:** Clinical course and outcomes.

Parameter^1^	Overall, *N* = 235^2^	HDU, *N* = 122^2^	ICU, *N* = 113^2^	*p*^3^
Length of hospital stay (days)	11 (7–20)	12 (5–22)	11 (8–16)	0.877
All-cause mortality	164 (69.7)	62 (50.1)	102 (90.2)	**<0.001**

1 HFNC, High Flow Nasal Cannula.

2 Median (Q1–Q3)/*n* (%).

3 Wilcoxon rank sum test; Pearson’s Chi-squared test.

**Figure 3:**
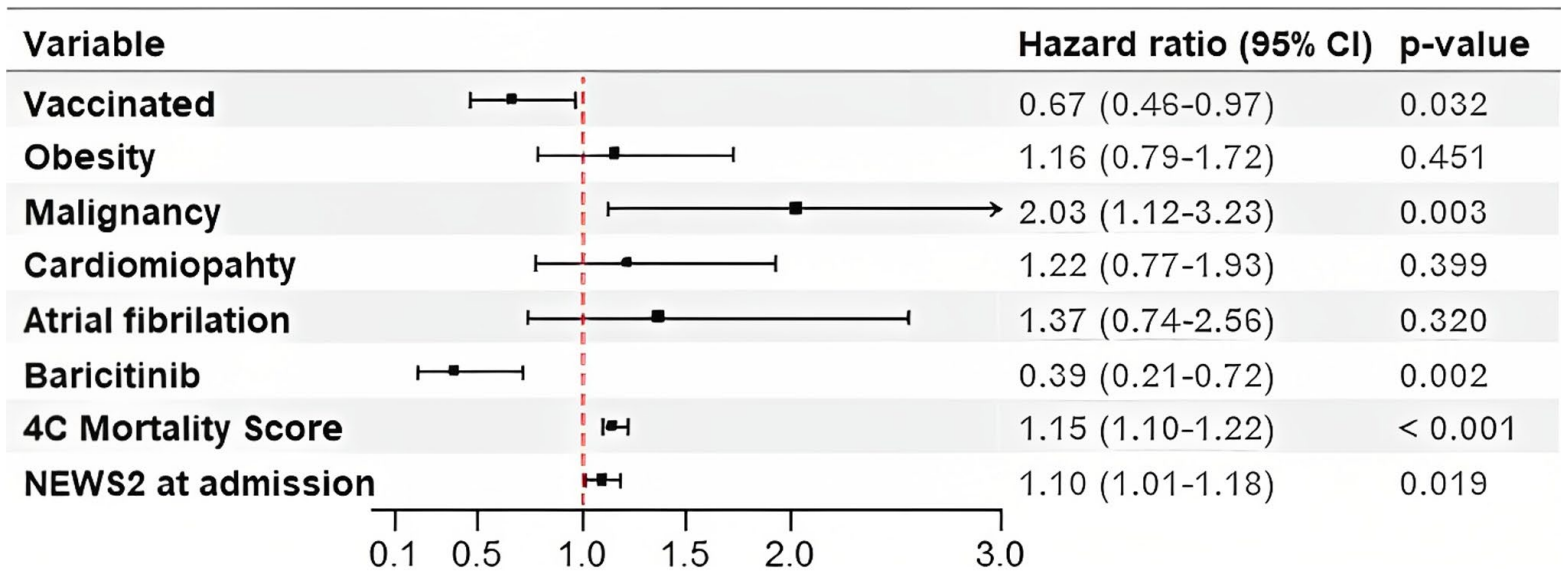
Hazard Ratios for the Secondary Outcome in patients with acute respiratory failure treated with High-Flow Nasal Cannula. 4C Mortality Score, Coronavirus Clinical Characterization Consortium Mortality Score; NEWS2, National Early Warning Score 2.

In the multivariable Cox proportional hazard analysis for the secondary outcome, vaccinal status (HR 0.7, 95% CI 0.49–1.02) and the intrahospital use of baricitinib (HR 0.43, 95% CI 0.23–0.79) emerged as the negative predictors of mortality. On the other hand, malignancy posed as the strongest positive mortality predictor with HR of 2.14 (95% CI 1.33–3.43). The significant indicators of the secondary outcome were also the higher levels of 4C mortality score and NEWS2 (Figure 3).

## 4. Discussion

Our retrospective study, focused on COVID-19 patients with AHRF that were treated with HFNC oxygen therapy, was performed during the fifth and sixth pandemic wave in Serbia with Delta and Omicron SARS-CoV-2 variants (22). The main findings in our study were as follows: (1) Our cohort consisted mostly of patients with the median age of 70 years, and hypertension as the most common comorbidity; (2) HFNC was successful in improving clinical course in 52% of patients; (3) The all-cause in-hospital mortality rate was high, reaching 70%, and it was significantly higher (90%) in patients with who experienced HFNC failure; (4) Patients treated with baricitinib showed significantly better survival; (5) Patients with concomitant malignant disease had a significantly worse prognosis. Previous retrospective studies at the beginning of the pandemic showed high mortality rates from 36% (23) among the older COVID-19 patients with AHRF who were treated with invasive mechanical ventilation up to the 62% as one Chinese study reported (24). As the pandemic progressed, the demand for ICU beds often exceeded hospitals’ capacities, leading to the conversion of non-ICU spaces to ICU units (25). The scarcity of ICU beds in many countries required alternative respiratory support for patients with AHRF, primarily focusing on HFNC, with the main goal being avoidance of intubation. The results the SOHO COVID trial (26) showed a clear advantage of HFNC to COT which was reflected in significantly lower intubation rate in the HFNC group compared to the COT group.

In our study, the majority of baseline characteristics, laboratory findings and oxygenation status were similar between two groups, except for the age in favor of the HDU group (72 vs. 70 years) and the disease duration prior to the diagnosis establishment with the median in ICU group being 5 vs. 4 days in the HDU group, *p* = 0.043. Similar findings have been reported in the cohort retrospective study from England from the beginning of the pandemic, where the timing of hospital admission was associated with poorer clinical outcomes in patients with COVID-19 (27). The most frequent comorbidities in our study group were hypertension, diabetes and obesity which is consistent with findings from other studies (28, 29). The patients in the ICU group were more obese, but without reaching the statistical significance. There were no significant differences regarding the baseline laboratory parameters, NEWS2, 4C mortality score and SaO_2_/FiO_2_ ratio between groups.

Among the individuals included in our study, 18.7% of them acquired intrahospital infections during their hospital stay with the incidence being significantly higher in the ICU group. Our results are similar with the results of the review of secondary pulmonary bacterial infections among the patients with COVID-19 pneumonia (30), showing the low incidence in general, but higher in ICU patients. In our study, the intrahospital pneumonia was observed exclusively in ICU group with higher prevalence of urinary tract infections (UTI). In the single-center retrospective study of COVID-19 patients during the first pandemic wave (31), secondary infections were present in 7.3% of times. The most common intrahospital infections were ventilator associated pneumonia (VAP) and UTI which is also comparable to our study. On the other hand, the European multicenter study focusing on VAP in patients that spent more than 48 h on mechanical ventilation reported slightly higher incidence of VAP, 36.1% (32). The absence of significant differences in baseline parameters and the frequency of corticosteroid and baricitinib usage suggests that it is reasonable to consider that the higher frequency of intrahospital infections in the ICU group could be due to a more rapid disease progression in these patients. The accelerated progression of the disease in the ICU group may have led to increased susceptibility to infections and the need for higher levels of respiratory support, ultimately contributing to the observed differences. Regarding the other analyzed clinical complications (thromboembolism, pneumothorax, and pneumomediastinum), no differences were reported among the groups.

The failure of non-invasive respiratory support that includes both HFNC and NIPPV, in patients with AHRF is directly related to delayed intubation and consequently higher mortality rate. Before the COVID-19 era, studies such as LUNG SAFE (33) demonstrated that the NIPPV in patients with severe AHRF and ARDS was linked to a significantly higher mortality rate compared to IMV (36.2% vs. 24.7%, respectively). FLORALI study (34), however, showed the advantage of HFNC utilization in comparison to the COT, where HFNC group exhibited significantly lower intubation rate. In the early phase of the COVID-19 pandemic, the recommendations were in favor of the early intubation, mostly due to the pronounced patient self-induced lung injury (P-SILI) and the higher mortality rate associated with the delayed intubation (35). Longer duration of the non-invasive respiratory support before the ICU admission has been identified as an independent risk factor of in-hospital mortality (36). Contrary to these findings, in our study, the overall duration of HFNC was 4 days, with a significant difference observed between HDU and ICU group (12 vs. 2 days, respectively, *p* < 0.001). Timely recognition of the non-invasive respiratory support failure is of the high importance. Studies have reported incidence of the NIPPV failure associated mortality ranging from 26.5% (37) to 49.6% (38). In our study, primary outcome, defined as the HFNC failure, was observed in 113 patients, most of whom (85%) initially received NIPPV prior to the ICU transfer and 78% of patients treated with NIPPV required subsequent mechanical ventilation.

Patients transferred to the ICU did not exhibit significant changes in CRP and PCT levels. However, significant increases were observed in D-dimer levels, as well as NLR and NEWS2 values. Additionally, there was a significant decrease in SaO2/FiO2 ratio, reflecting worsening oxygenation. The median ROX score at the ICU transfer was 3.40 (2.80–4.55). Our findings are comparable with the single-center study conducted by Talpoș et al. where the PaO_2_/FiO_2_ and ROX score emerged as the HFNC failure predictor (39).

The study from Jordan (40) reported a mortality rate of 23% during the early period of the pandemic, while Wuhan (41) reported a rate of 32%. Similarly, in a multicentre Italian study, which was also conducted during the early stages of the pandemic (36), the mortality rate increased to 43% following the NIPPV failure. Additionally, in New York (25), the mortality rate reached 61% after intubation. The overall in-hospital mortality rate in our study was high, reaching 70%, with a significantly more deaths reported in the ICU group (51% vs. 90%, *p* < 0.001, respectively). The survival analysis using Kaplan–Meier estimate reported the significantly better survival in the HDU group with a hazard ratio (HR) of 0.56 (95% CI 0.40–0.77, *p* < 0.001). The inclusion of severely ill patients with multiple comorbidities and a median age of 70 years could explain such a high mortality rate in our study group. The literature data shows that the progression to more severe COVID-19 disease is associated with higher age (≥50 years, with risk increasing substantially at ages over 65 years), race/ethnicity, as well as the presence of an underlying medical conditions (42–44).

The treatment protocols for COVID-19 have evolved during the pandemic, introducing new anti-inflammatory and antiviral drugs. Several randomized trials such as ACCT-2 and COV-BARRIER studies (45, 46) showed some promising results of baricitinib in terms of recovery time and reduction of mortality rates which is why the National Institutes of Health (NIH) Treatment Guidelines Panel has recommended the use of baricitinib in patients with rapidly increasing needs for higher respiratory support (13). The baricitinib was administered to 33 patients in our study, and 19 of them were successfully treated with HFNC. The use of baricitinib was associated with the better survival (HR 0.39, 95% CI 0.21–0.72) in the multivariate COX proportional analysis when adjusted for other covariates. These findings are consistent with the results of the COV-BARRIER study (46), A retrospective single-center study (47) comparing outcomes in patients treated with HFNC also reported a significantly lower 28-day all-cause mortality rate in patients treated with baricitinib compared to the standard care group. The vaccinal status is well known as a predictor of outcomes in patients with COVID 19 (48–50), and in our study, it also emerged as a significant negative predictor of mortality. We observed the similar vaccination rate between groups (27.2% in HDU vs. 28.7% in ICU group) and the vaccinated individuals had 30% reduced mortality rate compared to the non-vaccinated (HR 0.67, 95% CI 0.46–0.97). Similar results have been reported in a large retrospective study from the USA (49) where the proportion of unvaccinated individuals was also high (73.7%) with significantly higher mortality rate in unvaccinated group.

COVID 19 related mortality in patients with cancer is higher than in those without malignancy. According to a study on the impact of solid cancer on in-hospital mortality in COVID-19 patients, the 30-day in-hospital mortality rate was found to be higher in patients with solid cancer (31.7%) compared to those without cancer (20.0%) (51). Cancer not only serves as an independent risk factor for the COVID-19 disease severity (51), but also increases the risk of mortality (52). The International Severe Acute Respiratory and Emerging Infections Consortium (ISARIC) reported a significant difference in mortality rates between cancer and non-cancer patients (40.5% vs. 28.5%, respectively) (53). In our study, malignancy was observed to be the strongest positive predictor of intrahospital mortality with HR of 2.03 (95% CI 1.12–3.23). Additionally, the 4C Mortality Score and NEWS 2 score were identified as significant predictors of all-cause in-hospital mortality. The 4C score was proven to be one of the validated scoring systems for mortality prediction in COVID-19. It exhibits relatively high positive predictive value (62%) for mortality when it is over 15 (range 0–21) (53). In our study, the median 4C Mortality Score was 13 (10–15) in both groups and in a multivariable COX proportional analysis the HR of 1.15 (1.09–1.21) was observed. The median NEWS2 score at admission was also equal in both groups, 6 (4–8) vs. 6 (4–7), with the significant increase among patients transferred to the ICU, 8 (6–10), *p* < 0.001. A retrospective study from Romania (54) compared the predictive value of the 4C Mortality score, NEWS score, and CURB-65 score, with the NEWS score showing the best predictive power. In another study (55), the performance evaluation of NEWS and NEWS2 in predicting two outcomes (death and ICU admission) showed higher predictive power of scores for COVID-19 positive patients compared to those without COVID-19 detected.

This study has several limitations. Although the big number of patients’ data were processed, the study had a retrospective character and therefore, it possesses all of the limitations of a retrospective study. The study was also single-centered and we did not have a control group that could be used for a comparison. Finally, factors such as age, number of comorbidities, severity of the disease etc. could have had an impact on the abovementioned outcomes.

## 5. Conclusion

Our study is a contribution to the recommendations for application of HFNC in patients who fulfilled the criteria of severe COVID-19 pneumonia at the admission. The mortality rate in patients treated with HFNC failure is closely related to the comorbidity presence, clinical status of the patients and vaccinal status. In 52% of patients, HFNC was successful in treating AHRF in severe COVID-19 pneumonia.

## Data availability statement

The raw data supporting the conclusions of this article will be made available by the authors, without undue reservation.

## Ethics statement

The studies involving humans were approved by Ethics Committee of the University Clinical Centre of Vojvodina (protocol code: 00-39, date of approval: 9 February 2023). The studies were conducted in accordance with the local legislation and institutional requirements. The human samples used in this study were acquired from a by-product of routine care or industry. Written informed consent for participation was not required from the participants or the participants’ legal guardians/next of kin in accordance with the national legislation and institutional requirements.

## Author contributions

DO and APĐ contributed to the conceptualization and design of the study. DO, APĐ, and JŠ organized the database. SS-G, JŠ, MiĐ, ST, JB, MM, JJ, MP, IL, MlĐ, RBP, AV, and MJ collected the data. VĐ performed the statistical analysis. DO, VĐ, and AM wrote sections of the manuscript. AM conducted the supervision. All authors contributed to manuscript revision, read, and approved the submitted version.

## Conflict of interest

The authors declare that the research was conducted in the absence of any commercial or financial relationships that could be construed as a potential conflict of interest.

## Publisher’s note

All claims expressed in this article are solely those of the authors and do not necessarily represent those of their affiliated organizations, or those of the publisher, the editors and the reviewers. Any product that may be evaluated in this article, or claim that may be made by its manufacturer, is not guaranteed or endorsed by the publisher.
